# Sub-Liquid and Atmospheric Measurement Instrument To Autonomously Monitor the Biochemistry of Natural Aquatic Ecosystems

**DOI:** 10.1021/acsestwater.3c00082

**Published:** 2023-06-22

**Authors:** Miracle Israel Nazarious, Maria-Paz Zorzano, Javier Martin-Torres

**Affiliations:** † School of Geosciences, University of Aberdeen, Meston Building, King’s College, Aberdeen AB24 3UE, U.K.; ‡ Centro de Astrobiología (CAB), INTA-CSIC, Torrejon de Ardoz, 28850 Madrid, Spain; § Instituto Andaluz de Ciencias de la Tierra (CSIC-UGR), 18100 Granada, Spain

**Keywords:** aquatic ecosystems, liquid-atmosphere interaction, biogeochemical cycling, long-term measurements, autonomous monitoring

## Abstract

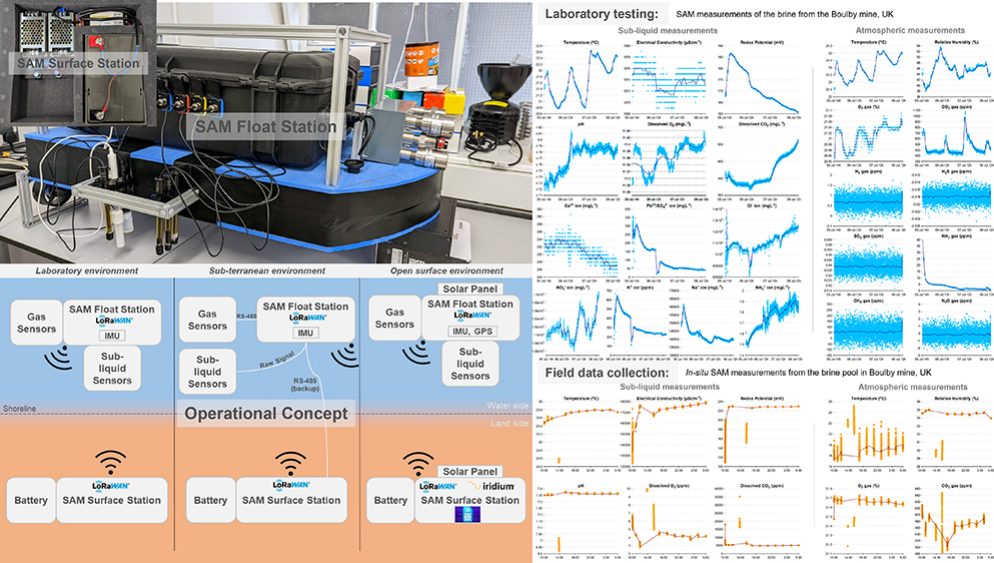

Monitoring the biochemistry of aquatic ecosystems is critical to understanding the biogeochemical cycling induced by microorganisms. They play a vital role in climate-gaseous drivers associated with natural ecosystems, such as methane emission in wetlands and peatlands; gas cycling and fixation: methane, sulfur, carbon, and nitrogen; water quality assessment and remediation; monitoring oxygen saturation due to contamination and algal proliferation; and many more. Microorganisms interact with these environments inducing diurnal and seasonal changes that have been, to date, poorly characterized. To aid with the long-term in-situ monitoring of natural aquatic ecosystems, we designed a Sub-liquid and Atmospheric Measurement (SAM) instrument. This floating platform can autonomously measure various sub-liquid and atmospheric parameters over a long time. This paper describes the design of SAM and illustrates how its long-term operation can produce critical information to complement other standard laboratory-based microbiological studies.

## Abstract 2

An autonomous instrument for real-time monitoring of life in water in extreme environments with potential planetary applications.

## Introduction

Microbial diversity is truly staggering.^1,2^ Microbial life has adapted to grow and proliferate by accessing the food and nutrients they need from the soil, aqueous, or aerial environments.^3^ Microbes are pivotal to all life on Earth because of their vast diversity in form and function.

Studies suggest that naturally occurring microorganisms in fresh and saltwater play diverse roles in ecosystems and are essential to Earth’s biogeochemical cycles.^4^ Depending on the aquatic environment, microorganisms in lentic systems can thrive in the littoral zone,^5^ a region of a liquid body near the shoreline, well-lighted, shallow, and warmer than other regions of the water; limnetic zone,^6^ further away from the shore, colder and having sunlight only in the upper 100 feet or so; benthic zone,^7^ at depths without any oxygen and sunlight; subterranean systems,^8^ with varied salt concentrations and pH levels (acidophiles and alkaliphiles); salty water bodies,^9^ with higher salt concentration, (halophiles), higher pH, and lower nutrients; and hydrothermal vents^10^ and within biofilms,^11^ a grouping of cells surrounded by a polymer matrix providing a protective barrier to the external environment. The activity in these ecosystems may also vary seasonally, and by monitoring them continuously over a long time, a detailed account of the nutrient and gas cycling can be established.^12^ The interplay between these ecosystems, the atmosphere, the fixation of nitrogen, and the release of carbon dioxide and methane, has not been systematically characterized, partially due to the technological difficulties in the instrumentation for the long-term monitoring within aquatic systems.

In order to study these processes, previous research has focused on monitoring the physicochemical parameters at short timescales in the order of days at most. Various autonomous platforms have also been developed, each measuring parameters to investigate microbial activity in their respective ecosystems. Table 1 summarizes a review of the recent studies on the different measurements used to understand various aquatic ecosystems.

**Table 1:** Summary of Past Studies in Aquatic Ecosystems

reference	study location	scientific objectives	measurements	sensors	sensor specifications
Chang et al.^13^	Chia-Ming Lake, Taichung, Taiwan	water quality monitoring	pH	SEN0161 pH sampling probe	operational temperature: 0–60 °C
	(Lat: 23.2934, Long: 121.0341)				measurement range:
	Jan 15–18, 2019 (test duration: 4 days)				0–14 (±0.1 @ 25 °C)
Lindborg et at.^14^	Lake catchment, Kangerlussuaq, West Greenland	in situ measurements to study the present-day periglacial processes	pH, temperature, dissolved oxygen, and alkalinity	TROLL 9500 multi-parameter probe	operational temperature: −5–50 °C
	(Lat: 67.1259, Long: −50.1803)				measurement range:
	June 15, 2013 – August 23, 2014 (test duration: 1 year and 3 months)				pH: 0–12 (accuracy: ±0.1; resolution: 0.01)
					*T*: −5–50 °C (accuracy: ±0.1 °C; resolution: 0.01 °C)
					DO: 0–20 mg/L (accuracy: ±0.2 mg/L; resolution: 0.01 mg/L)
					EC: 5–112,000 μS/cm (accuracy: ±0.5% or 2 μS/cm)
			dissolved oxygen and temperature	HOBO U26-001	operational temperature: 0–50 °C
					measurement range:
					DO: 0–30 mg/L (accuracy: ±0.2 mg/L up to 8 mg/L and ±0.2 mg/L from 8–20 mg/L, resolution: 0.02 mg/L)
					*T*: −5–40 °C (accuracy: ±0.2 °C, resolution: 0.02 °C)
			water temperature at 4.3 m depth	Level TROLL 700 transducers	operational temperature: −20–80 °C
					measurement range:
					*T*: −5–50 °C (accuracy: ± 0.1 °C, resolution: 0.01 °C)
Eugster et al.^15^	Toolik Lake, Alaska	water-atmosphere fluxes of carbon dioxide, water vapor, and energy	turbulence	Eddy Covariance systems:	operating frequency: 10 and 20 Hz
	(Lat: 68.6322, Long: −149.606)			Applied Technologies, model SAT-211/3Vx ATI 3-D sonic anemometer and thermometer	
	July 27–31, 1995 (test duration: 5 days)				
	Soppensee, Lucerne, Switzerland				
	(Lat: 47.0904, Long: 8.0803)				
	September 21–23, 1998 (test duration: 3 days)			Gill Instruments Solent HS 3-D Research grade sonic anemometer and thermometer	operating frequency: 100 Hz
					resolution: 20 Hz
			carbon dioxide and water vapor concentration	LI-COR 6262 Infrared Gas Analyzer	absolute mode
			air temperature and moisture at Eddy Covariance height	HMP35C Väisälä-type thermometer/hygrometer	operational temperature: −35–60 °C
					measurement range:
					*T*: −35–55 °C (accuracy: ±0.2 °C for 0–60 °C, ±0.4 °C for −24–48 °C and 0.9 °C for −38–53 °C)
					RH: 0–100% (accuracy: ±2% for 0–90% and ±3% for 90–100%)
			lake surface, air temperature, and moisture	home-built aspirated psychrometer with AD592 integrated circuit temperature sensor	measurement range:
					–25–105 °C
			wind profile	TSI Model 8470 hot wire anemometers	measurement range:
					0–5 m s^–1^ (accuracy: ±3%)
			net radiation	Fritschen-type REBS Model Q*6 net radiometer	
				Davos-type Swissteco model S-1 net pyrradiometer	
			water temperature	REBS Model STP-1 Platinum resistor sensors	
			temperature at depths of 0.5, 2.5, 4.5, 6, 7, 8, 9, 10, 12, 17, and 25 m	Richard Brancker Research TR-1000 Thermistors	accuracy: ±0.05 °C, resolution: 0.002 °C
				SeaBird SBE 9/11 CTD profiler with FP-07 Fast response temperature probe	response time: 15 ms
					resolution: 0.1 m K
			water velocities between 3.3 and 7.2 m depth	Nortek Signature 500 Acoustic Doppler Current Profiler	operating frequency: 1.5 MHz
					resolution 0.1 mm s^–1^
Cryer et al.^16^	Belize River, Belize City	investigate coastal ocean acidification in shallow environments	conductivity	RBR inductive current and receiver	accuracy: ±0.003 mS/cm
			temperature	RBR aged glass thermistor	accuracy: ±0.002 °C
			depth		accuracy: ±0.05%
	(Lat: 17.4576, Long: −88.6398)		dissolved oxygen	RBR, normal foil	accuracy: ±8 μM
			partial pressure of carbon dioxide (pCO_2_)	Turner C-sense NDIR detector	accuracy: ±3%
					resolution: 1000 ppm
			pH	Idronaut Glass membrane pH electrode	accuracy: 0.01
	November 24–26, 2018 (2.5 days)		nitrate	Seabird SUNA V2 Optical UV	accuracy: ±8 μM
			chlorophyll fluorescence	Sea-Bird WET Labs EcoPuck Triplet Optical Sensor	fluorescence
					(695 nm)
					accuracy: ±0.025 μg/L
			colored dissolved organic matter		fluorescence
					(460 nm)
					accuracy: ±0.28 ppb
	October 17–20, 2019 (test duration: 4 days)		optical backscatter		optical backscatter
					(700 nm)
					accuracy: ±0.003 m^–1^
			dissolved organic carbon, turbidity, total organic carbon	S::can Spectro::lyser	accuracy: ±2%
				UV–vis spectrometry	
				(190–750 nm)	
			current profiling	Nortek Signature 500 Acoustic Doppler Current Profiler	accuracy: ±0.1 cm/s (velocity resolution)
Cao et al.^17^	aquaculture zone near Goazhou, China	three-dimensional water quality monitoring at different depth levels	pH	Telesky	measurement range: 0–14 accuracy: ±0.7%
	(Lat: 21.8811, Long: 110.8427)				
	test duration: 4100 s		total dissolved solids	Waaax	measurement range: 0–1000 ppm
					accuracy: ±5%
			turbidity	Eixpsy	measurement range: 0–4000 NTU
					accuracy: ±0.75%
Awomeso et al.^18^	Ogun River, Nigeria	assess the water quality and permissible limits in drinking water	temperature, pH, total dissolved solids, electrical conductivity	Combo HI 98130 combined temperature/pH/TDS/EC meter	operational temperature: 0–50 °C
					measurement range:
					*T*: 0–60 °C (accuracy: ±0.5 °C, resolution: 0.1 °C)
					pH: 0–14 (accuracy: ±0.01, resolution: 0.01)
	(Lat: 6.8897, Long: 3.3804)				EC: 0–20 mS/cm (accuracy: ±2%, resolution: 0.01 mS/cm)
	April 2013–January 2014 (test duration: 10 months)				TDS: 0–10 ppt (accuracy: ±2%, resolution: 0.01 ppt
			dissolved oxygen	HachsensION DO meter	operational temperature: 0–50 °C
					measurement range: 0–22 mg/L
					accuracy: ±0.5%
					resolution: 0.01 mg/L
			color, turbidity,	Hach DR/4000 UV–visible spectrophotometer	wavelength range: 190 to 1100 nm
					accuracy: ±1 nm
			total suspended solids	gravimetrically	
			total solids = total dissolved solids + total suspended solids		
			anions (F^–^, Cl^–^, PO_4_^3–^, and NO_3_^–^)	chloride (Cl^–^): Mohr’s silver nitrate method; dissolved silica (SiO_2_): heteropoly blue method; nitrate (NO_3_^–^): sodium salicylate method; sulfate (SO_4_^2–^) and fluoride (F^–^): spectrophotometer	
			metals (Fe, Pb, Cd, Zn, Na, and K)	Fe, Pb, Cd, Zn: Buck Scientific Model 200 atomic absorption spectrophotometer	wavelength range: 190 to 900 nm
					accuracy: ±0.2 nm
				Na and K: Jenway Model 970 Flame Photometer	
Ryu^19^	Parkcenter Park, Boise, Idaho USA	real-time monitoring and visualization of water quality to advance environmental research activities, especially for impaired waterways (e.g., lakes, rivers, and reservoirs)	dissolved oxygen	Atlas Scientific Wi-Fi Hydroponics Kit	measurement range: 0–20 mg/L
			electrical conductivity		measurement range: 0–200,000 μS/cm
					resolution: 0.01 μS/cm
	(Lat: 43.5978, Long: −116.181)		pH		measurement range: 0–14
					resolution: 0.01
	test duration: 20 min		water temperature		measurement range: −200–850 °C
					accuracy: ± (0.15 + (0.002 × *t*))
Rao et al.^20^	laboratory	continuous measurements of biologically relevant physicochemical parameters in freshwater to provide insights into the current status of changing water conditions and assist in identifying pollution sources	pH	Phidgets Model 3550_0 - A5P200–2-1 M-BNC pH Lab Electrode	operational temperature: 0–80 °C
					measurement range: 0–14
	test duration: 8000 s		light	Phidgets Model 1127_0 Precision Light Sensor	measurement range: 0–1000 lx
			temperature	Atlas Scientific ENV-TMP Field Ready Temperature Sensor	measurement range: −20–133 °C (accuracy: ±1 °C)
			electrical conductivity	Atlas Scientific Conductivity Sensor	measurement range: 1300–40,000 μS
			dissolved oxygen	Atlas Scientific DO Sensor	measurement range: 0–20 mg/L
			oxidation–reduction potential	Atlas Scientific ORP Sensor	measurement range: ±2000 mV
De Vito-Francesco et al.^21^	New Danube River, City of Vienna	on-site detection of heavy metal pollution plumes in surface water	heavy metals: Pb and Cu	Pb: Hach Lange Model LCK306	measurement range:
	(Lat: 48.2211, Long: 16.4304)			Cu: Hach Lange Model LCK529	Pb: 0.1–2.0 mg/L
	test duration: 2 h				Cu: 0.01–1.0 mg/L and 40–400 μg/L

Although the past studies were able to collect a good amount of data on various natural aquatic ecosystems, they lack consistent long-term field observations (except for a test duration of 1 year and 3 months by Lindborg et al.^14^ and multiple point observations over 10 months by Awomeso et al.,^18^ all other tests were short term) on micro-scale temporal variations. Hence, they could not provide a detailed account of the biogeochemical liquid–gas phase interchanges and cross-talks in niche ecosystems over several months. This is the same case with most field campaigns, where manual single time point measurements in logistically limited one or few locations are considered to conclude the complex biogeochemical processes that occur in the order of days, weeks, or even months.

To address this, we designed a maneuverable floating observatory capable of performing autonomous long-term measurements in remote field environments leveraging sensor technologies that do not require frequent calibration or maintenance and operate with low power, mass, and data budget. This approach comes with its associated engineering challenges in terms of power, data communication, portability, durability, robustness, and environmental compatibility.

Monitoring the physicochemical parameters of the liquid body, such as temperature, pH, electrical conductivity, oxygen reduction potential, dissolved oxygen, turbidity, salinity, total dissolved solids, and atmospheric parameters such as temperature, pressure, relative humidity, wind speed, wind direction, have been generally implemented to assess the water quality to study the water-atmosphere fluxes of CO_2_^15^ and coastal ocean acidification in shallow environments,^16^ evaluate the permissible limits for human and animal consumption and agricultural uses,^18,22,23^ monitor cyanobacterial harmful algal blooms,^24^ and conduct field investigations for highly polluted and/or shallow high-risk waters.^25^ This has several implications if the quality of our drinking water, agriculture and aquaculture, and biochemical exchanges induced by microorganisms in aquatic ecosystems is monitored systematically.

Such systems can also be used for long-term monitoring of the climate-impacting processes associated with natural ecosystems, such as methane emission in wetlands and peatlands; gas cycling and fixation: methane, sulfur, carbon, and nitrogen; water quality assessment and remediation; and monitoring oxygen saturation due to contamination and algal proliferation, in remote locations with restricted access to power, data communication, and maintenance.

## Methods

### Science Goals and Objectives

The design of SAM has been done taking a diverse collection of natural aquatic environments as possible targets of study, such as the natural brine pool in the Boulby Mine, UK;^26^ subterranean sulfidic lake in the Movile Cave, Romania;^27,28^ hypersaline Tso Kar Lake in Ladakh, India;^29^ anoxic, sub-zero hypersaline high arctic spring in Nunavut, Canada;^30^ and subglacial Lake Bonney in Antarctica.^31^ The scientific goal of the SAM instrument is to monitor the various physicochemical parameters in the liquid and various gas concentrations in the aquatic ecosystem’s atmosphere to help determine the biogeochemical cycles—exchange and cross-talk between liquid and gas phases induced by microbes.

The following are the primary design requirements of the instrument:

i. It must be able to perform continuous, autonomous, and pre-programmable, long-term (up to a year) measurements of various selected parameters, namely, environmental, electrochemical, ionic, and dissolved gas concentrations and atmospheric gas concentrations.
ii. It must be able to measure auxiliary data about the environment such as localization and light condition.
iii. It should be able to navigate and position at a particular region of interest for measurement.
iv. It should store the data within its memory until retrieval and have the option to send data to the shore up to at least 5 kilometers using long-range (LoRA) WAN network or remotely to a server through a GSM cellular or Iridium satellite network.
v. It should be built using commercial off-the-shelf components with state-of-the-art technology.

### Functional Requirements: Payload

Table 2 specifies the requirements in terms of the measured parameters and their measurement range, with the accuracy requirement of each measurement given in brackets. These requirements were built on the choice of measurements and their ranges used in the existing literature and the best commercially available sensors. In general, all the selected probes and sensors are required to have a lifetime of at least a year between maintenance while measuring at an hourly frequency.

**Table 2:** Payload Requirement Traceability Matrix

measurement objectives	measurement requirements	sensor	minimum sensor requirements
environmental	sub-liquid and atmospheric temperature (*T*), pressure (*P*), and relative humidity (RH) over the aquatic ecosystem	environmental probes are to be submerged in the liquid, and environmental sensors are to be suspended in the atmosphere	operational temperature: 1–40 °C
			measurement range: *T*: 1–40 °C (±0.1 °C); *P*: 100,000–150,000 Pa (±2%); RH: 0–100% (±2%)
electrochemical	sub-liquid electrical conductivity (EC), oxidation–reduction potential (ORP), pH of the liquid	electrochemical probes to be submerged in the liquid	operational temperature: 1–40 °C
			measurement range: EC: 0–200,000 μS/cm (±2%); ORP: −1020–1020 mV (±1 mV); pH: 0–14 (±0.002)
ion concentration	cations and anions in the aquatic ecosystem significant for microbial metabolism including the heavy ions	ion-selective electrodes (ISE) are to be submerged in the liquid measuring cations and anions	operational temperature: 1–40 °C
dissolved gas concentration	concentrations of dissolved oxygen (DO), carbon dioxide (DCO_2_), and ammonia (DNH_3_)...in the liquid	ISE to be submerged in the liquid measuring dissolved gases	operational temperature: 1–40 °C
			measurement range: DO: 0.1–200 + % (±1 mV); DCO_2_: 0–400 ppm (±1 mV); DNH_3_: 0.02–17,000 mg/L
atmospheric gas composition	concentrations of atmospheric oxygen (O_2_), carbon dioxide (CO_2_), methane (CH_4_), nitrous oxide (N_2_O), hydrogen (H_2_), sulfur gases (hydrogen sulfide, H_2_S, and sulfur dioxide, SO_2_), ammonia (NH_3_) over the aquatic ecosystem	atmospheric gas sensors to be suspended over the aquatic ecosystem	operational temperature: 1–40 °C
			measurement range: O_2_: 0–30%; CO_2_: 0–5%; CH_4_: 0–5%; SO_2_: 0–2000 ppm; H_2_S: 0–2000 ppm; H_2_: 0–5000 ppm; NH_3_: 0–100 ppm; N_2_O: 0–1%

Table 3 specifies the requirements for the platform systems that are responsible for their floatability and maneuverability, power and data management, and communication protocols suitable under various types of deployments and operational configurations. The descriptions of the terminologies used in this section, namely, ″float station″ and ″surface station″ are elaborated under the sections “Design of the SAM instrument” and “Design of the floating platform” further in this paper. The requirements mentioned here take a conservative approach to enable the science goals and objectives of the SAM instrument so that at least the minimum amount of data is gathered for a particular study area of interest. This approach also helps keep the instrument’s costs and risks at the lowest possible level. Significantly for communication, various short, medium, and LoRA protocols were explored to add sufficient redundancy if the primary communication link fails.

**Table 3:** Platform Requirement Traceability Matrix

system objectives	system requirements	possible solutions	minimum specifications
floatation	to float on the liquid body while carrying the payload and other systems used for power and data management, localization, and navigation	lightweight foam	minimum weight of payload and other systems: 10 kg
power supply	to generate and supply power to the payload and other systems	solar panel	minimum 50 W high efficiency solar panel
power distribution	to efficiently direct the generated solar power to power the payload and other systems; and battery for storage	solar charge controller and DC converter	high efficiency 12 V solar charge controller, 12 → 5 V DC and 12 → 3.3 V DC converters
power storage	to provide backup power for the payload and other systems	battery	minimum 30 Ah lead acid AGM battery
communication	protocol to handle command and data requests	I^2^C, RS-485, LoRA WAN, GSM and iridium network	short-range (0–30 cm) I^2^C communication between sensors to the main computer, LoRA (30 cm–1200 m) RS-485 communication between sensors with an extended cable to the main computer, wireless LoRa WAN communication (0–5 km) between float station main computer and surface station main computer, GSM or iridium network (hundreds and thousands of km) for remote access of data to the main computer of the surface station
data acquisition	to send commands to individual sensors and return data or error code	Arduino microcontroller	Arduino MKR family microcontroller with support for I^2^C, RS-485 protocols and LoRA WAN, GSM, and iridium network
data storage	to store the received data until retrieval	MicroSD	1GB MicroSD card at the float and surface station main computers
localization	to monitor the movement of the instrument in the liquid body	absolute orientation sensor	inertial measuring unit measuring three-dimensional acceleration, yaw rate, and magnetic field strength data each in three perpendicular axes.
navigation/propulsion	to move the instrument in the liquid body and position	thruster	two underwater brushless DC electric motor thrusters, one on each side of the floating platform at the rear end, for forward and backward propulsion, and differential steering
payload deployment	to deploy the payload in the liquid body before starting the measurements	motor	two waterproof brushless DC servo motors, one on each side of the floating platform
auxiliary measurement	to measure additional parameters about the environmental conditions around the instrument circuits	environmental sensor	temperature, pressure, relative humidity, light intensity within the enclosure of the instrument

### Functional Requirements: Floating Platform

In addition to the functional requirements mentioned in Tables 1 and 2, the SAM instrument has been designed to be portable for easing logistics during field campaigns and made durable, robust, and environmentally compatible. The choice of materials, from the floatation systems of the SAM instrument to its sensors, has been carefully designed considering the harsh environment of its application field sites, for example, the corrosive salty environment in the Boulby Mine, UK, or the corrosive sulfur gas-rich environment in the Movile cave, Romania. The potential impacts of the corrosive nature of the environments will be investigated in detail during their field deployments.

### Sensors and Measurements

Following the justification provided to monitor various sub-liquid and atmospheric parameters that are relevant and scientifically meaningful and comply with the scientific goals and objectives of the SAM instrument, Table 4 introduces the various physicochemical parameters in the liquid and various gas concentrations in the atmosphere of the aquatic ecosystem to be monitored by the SAM instrument. All the selected sub-liquid and gas sensors are commercially ava1ilable from their standard performance catalogue, while for gas sensors, the measurement range and resolution were custom-ordered to suit the range of values we would expect during the laboratory and field deployments of interest. The gas sensors also can adjust the range (narrow or wide band measurements; resolution varies depending on the selected range) as required before implementing the sensors for measurements.

**Table 4:**
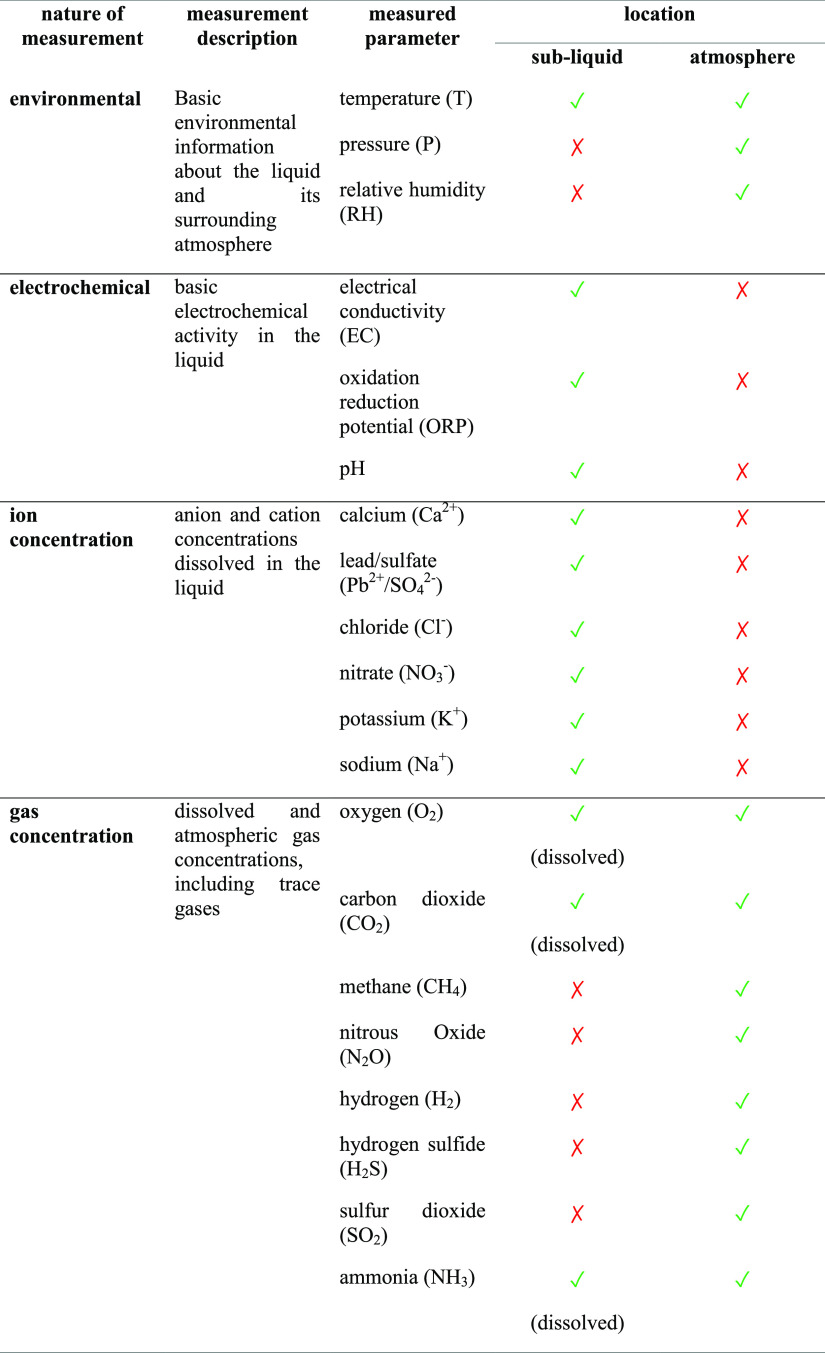
Suite of Sensors Used in the SAM Instrument, where 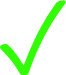 Is the Availability of the Sensor in that Location and 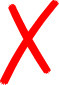 Represents the Unavailability of the Sensor

### Sensor Calibration

The 13 EZO circuits used for sub-liquid sensors are first calibrated using standard solutions for each of their corresponding measurements in the range of values suitable for the deployed location and their expected range of measurements. As a starting point, the expected range of measurements is estimated from the previous literature or with short-term data collection from the samples, if available in the laboratory. The calibration procedure is carried out in ambient laboratory settings. The calibration standards (Atlas Scientific and Hanna Instruments) are carefully chosen to cover the expected range of measurements. The temperature circuit is calibrated to one point for room temperature at 23 °C, using a commercial RS-91 mini temperature and relative humidity data logger. The electrical conductivity circuit is calibrated for an application in a brine environment with 12,880 and 150,000 μS/cm conductivity standards as low and high calibration points, respectively. The calibration point with the dry electrode is used as the zero-offset measurement. The pH circuit is calibrated for the entire range (0–14) with three calibration points at 4.00, 7.00, and 14.00 with their respective standards. The dissolved oxygen circuit is calibrated for zero dissolved oxygen concentration with the 0 mg/L standard. The oxidation–reduction potential circuits that are used for ORP measurements and for other measurements for dissolved carbon dioxide, ions such as calcium, lead/sulfate, chloride, nitrate, potassium, sodium, and ammonia, are calibrated to one point with the 225 mV ORP standard. All the gas sensors and relative humidity sensors are factory-calibrated.

### Floating Platform Design

**Figure 1:**
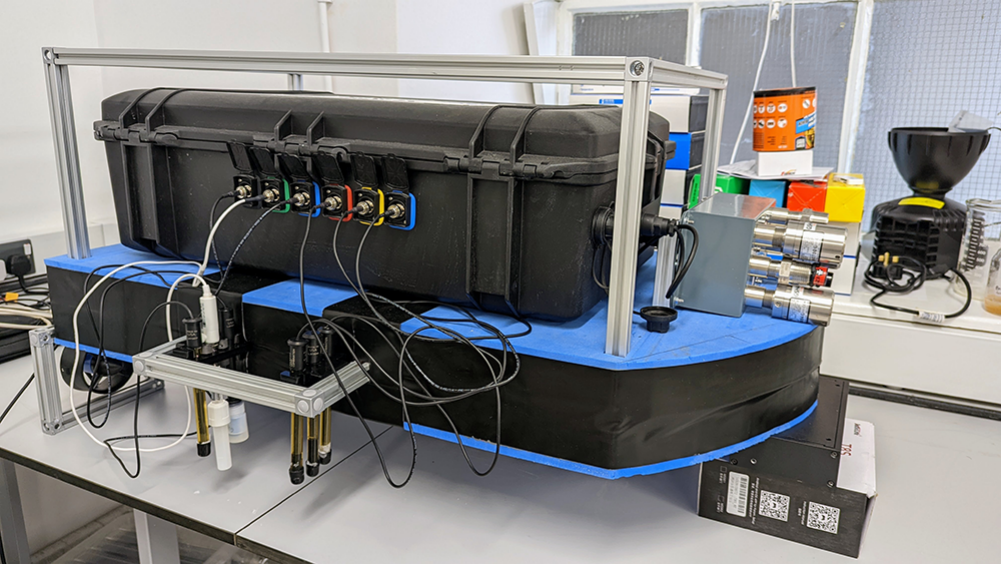
SAM instrument fitted with the floating platform and sub-liquid sensors (in the front of the image) and gas sensors (on the right-hand side of the image).

Per the primary design requirements of the SAM instrument and the requirements specified in Table 3 for the platform systems concerning the floatability and maneuverability, power and data management, and communication protocols suitable under various types of deployments and operational configurations, the platform systems documented in the section were carefully designed. Figure 1 shows the SAM instrument mounted on the floating platform with its primary and auxiliary systems.

#### Primary Systems

The most important prerequisite for the SAM instrument, besides floating to allow for relevant liquid and atmospheric gas measurements to achieve its scientific goals and objectives, is to enable a continuous supply of power, to retrieve and store data safely and reliably, and to navigate the instrument to selected areas of interest in the liquid body.

A 60 W solar panel (Mobile Solar Chargers) is chosen to be the suitable solution for a continuous power source which is further regulated through a solar charge controller (Mobile Solar Chargers) before charging the 200 W, 52 Ah lithium-ion battery (Mobile Solar Chargers) that is housed in the float station (placed on the floating platform). A backup power source is included in the surface station (placed in the shore of the liquid body) with a commercial 35 Ah lead acid AGM battery (typically used in cars), which is further regulated with a maximum power point tracking solar charge controller (GV-5 Lead Acid 12 Volt MPPT, Genasun). The Li-ion battery in the surface station may be sourced from an additional 60 W solar panel (Mobile Solar Chargers). The float station’s primary battery and the surface station’s backup battery are connected in parallel to provide a uniform 12 V DC power supply to the 12 → 5 V DC converter that is supplied to the sensors and circuits in the instrument.

The data communication protocol between the sensors and the microcontroller (Arduino MKR WAN 1310) is selected depending on the distance between the designated location of the sensors during deployment and the microcontroller. All 13 sub-liquid sensors are within a 30 cm range of the microcontroller. Hence, the I^2^C communication protocol is used, while the data communication protocol for gas sensors varies. Depending on the operational configuration and location of gas sensors during deployment, either I^2^C (short range, 0–30 cm) or RS-485 (long range, 30 cm–1200 m). The data that are retrieved from the sensors to the microcontroller are stored in a 1 GB MicroSD card in the float station. A copy of the data is transmitted through the LoRa WAN network established between the float and surface stations for easier retrieval of data from the 1 GB MicroSD card installed in the microcontroller (Arduino MKR WAN 1310) in the surface station. More information on the data communication protocol and data transfer and storage is provided under the “Operational Concept” section.

Two T200 thrusters (Blue Robotics), one on either side, are used for navigating the floating platform along with the float station in a liquid body. By operating both the thrusters in one direction, the forward and reverse motion can be achieved, while the speed of rotation of the rotors will decide the speed of motion, and by operating the thrusters in opposite directions, steering is achieved. The speed and steering of the thrusters are controlled with a thruster commander (Blue Robotics) linked with basic electronic speed control (Blue Robotics) for each thruster, all of which are powered by an individual 14.8 V, 15.6 Ah Lithium-ion battery (Blue Robotics).

#### Auxiliary Systems

These are the optional systems that complement the measurements and operation of the SAM instrument. They comprise systems responsible for localization, payload deployment, and float station environmental measurements.

An inertial measuring unit (Arduino MKR IMU Shield) based on the absolute orientation sensor (BNO055, Bosch Sensortec GmbH) is used for localization to monitor the movement of the instrument in the liquid body, measuring the acceleration, yaw rate, and magnetic field strength data each in three perpendicular axes. These data will complement the liquid and gas measurements and enable mapping the measurements with respect to the location in the liquid body.

The diverse collection of natural aquatic environments chosen as possible targets of study with the SAM instrument poses challenges to sensor performance and maintenance during long-term deployments. Due to the corrosive and reactive nature of various aquatic systems, limiting the sensors’ exposure to the environment is beneficial. Particularly for the sub-liquid sensors, which are ideally submerged in the liquid body for the entire deployment duration, the chances of corrosion and damage to the sensor body are very high. In order to counter that, a payload array deployment system is proposed and implemented. The system uses two waterproof brushless DC servo motors, one on each side of the floating platform, connected to a 3D printed holder for the 13 sub-liquid sensors, which will be deployed into the liquid body just before starting the measurements.

To monitor the health of the vital systems in the float station, an environmental sensor is installed to measure the temperature, pressure, relative humidity, and light intensity within the enclosure of the float station. Table 5 shows a summary of the capabilities and the final design implemented in the primary and auxiliary systems of the floating platform.

**Table 5:**
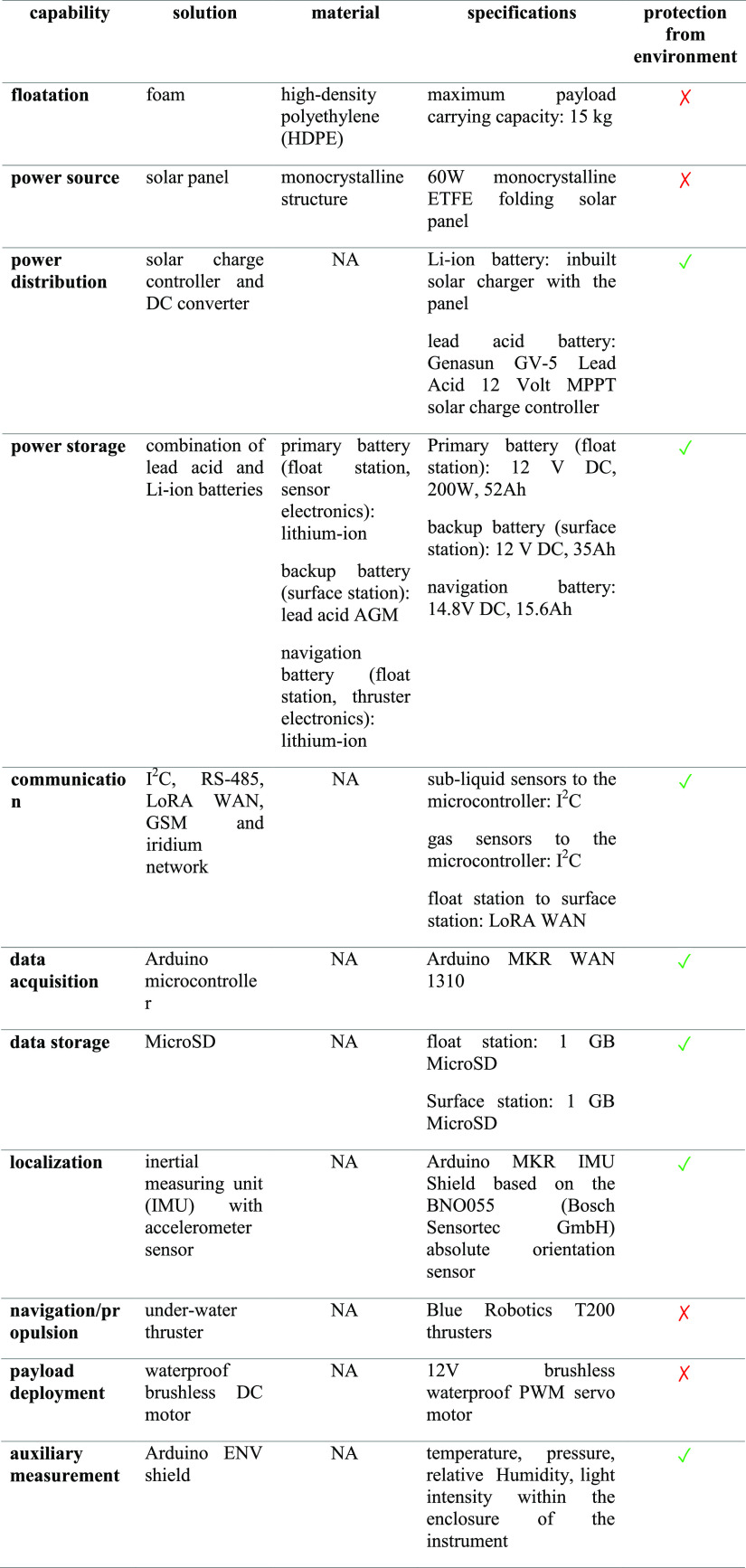
Summary of the Floating Platform Capabilities

The final design of the SAM instrument including the payload (sub-liquid and gas sensors and their supporting circuits) and floating platform has dimensions of 1045 mm length × 795 mm width × 525 mm height, a mass of 14.9 kg, and maximum power consumption of ∼2.5 and ∼209.5 W with thrusters.

### Operational Concept

**Figure 2:**
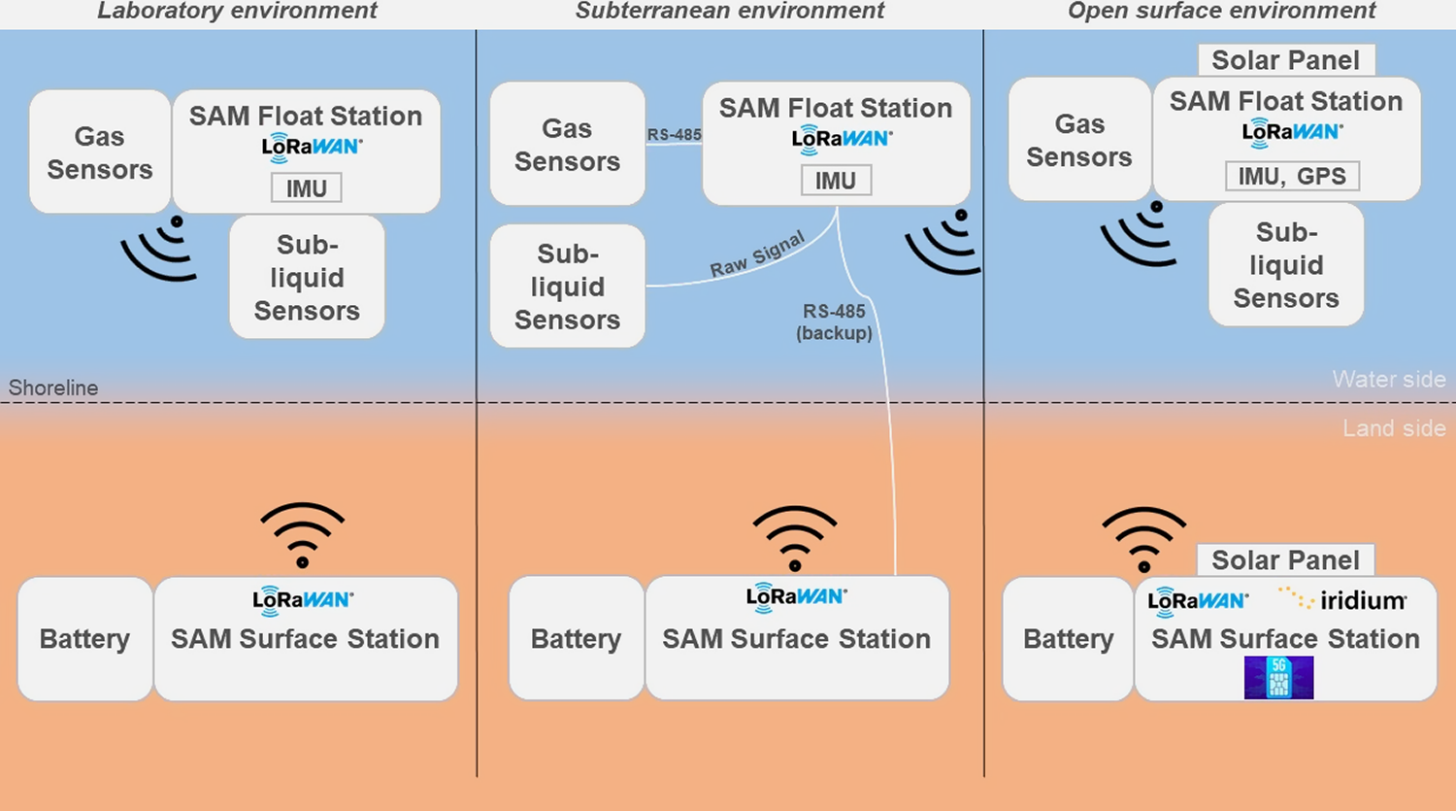
Operational concept of the SAM instrument depicting the various mission scenarios and their associated system configurations.

This section discusses the mission scenarios during the deployment of the SAM instrument in various environments, namely, laboratory, sub-terranean, and open surfaces. Figure 2 shows the operational configurations of the SAM float station and surface station concerning the deployment of sensors, power source (solar panel/battery), communication protocols between the stations, and localization.

#### Laboratory Environment

With minimal requirements for power due to unlimited supply from the common laboratory power grid, there is no reliance on solar panels as a power source in this configuration. The primary and backup batteries may nominally be used in this configuration without a cable for a direct power supply. The deployment of the sub-liquid and gas sensors are straight forward since there are no special cases to separate the sensors from the float station unless testing for a mission scenario. So, the I^2^C data communication protocol is used for all the sensor data retrieval. The data are reliably transmitted through the LoRa WAN network from the microcontroller in the float station to the microcontroller in the surface station or other data-archiving hardware for long-term data storage in laboratory servers. There are also other provisions for data transfer through the internet. Due to restricted access to GPS within the laboratory buildings, an inertial measuring unit sensor is used for localization in case of deployments in an aquatic pool inside a laboratory environment.

#### Subterranean Aqueous Environment

In this configuration, due to the nature of the environment, which is typically remote and deep underground without sufficient infrastructure, there is no source of power from the grid or using a long cable from a solar panel, no access to GPS for localization, no access to the internet, cellular, or satellite communication for data transfer. So, the batteries seem to be the only choice, and because of that, the duration of the deployment is limited unless the battery in the surface station is replaced with a fully charged one between measurements. Also, depending on the relevance of the measurement, the sub-liquid and gas sensors may have to be separated from the float station with a longer cable that makes the I^2^C data communication unreliable, especially for the gas sensors which send the I^2^C clock and data signals (sub-liquid sensors send raw analog signals and will have to be recalibrated with longer cables). For this reason, the data communication protocol between the gas sensors and the float station is replaced with the RS-485 data communication (Arduino MKR 485 Shield), which is more reliable for data transfer over long cables up to 1200 m. Typically for SAM instrument deployment, we anticipate a maximum cable length of 10 m between the sensors (sub-liquid and gas) and the float station. The data are transmitted between the float and surface stations through the LoRa WAN network, and the data are stored in their respective MicroSD cards until retrieval, since there is no provision to transfer them to laboratory servers through the internet or cellular or satellite networks. In the case of a cave environment with many turns and walls, the LoRa WAN data communication protocol may not seem effective, and there may be a loss of data transmitted between the float and surface stations. In that case, an RS-485 data communication link is established between the stations as a backup data transfer link. Also, due to restricted access to GPS within the subterranean environment, an inertial measuring unit sensor is used for localization.

#### Open Surface Aqueous Environment

In this configuration, there is no limitation for the power source from the solar panel, unrestricted access to GPS for localization, unrestricted access to wired or wireless internet, and cellular and satellite data communication networks. The default configuration of the SAM instrument with its solar panel is used as a primary power source charging the primary battery housed within the float station, while another solar panel charging the battery within the surface station may serve as a backup during the night. The sub-liquid and gas sensors are in proximity to the float station; hence, the I^2^C data communication protocol is reliably implemented for data transfer between the sensors and the microcontroller in the float station. The data are also transmitted between the float and surface stations through the LoRa WAN network, and the data are stored in their respective MicroSD cards until retrieval. There is also a provision to transfer the data from the surface station to laboratory servers through the internet or GSM cellular or iridium satellite networks, all of which have commercial shields supported for the microcontroller used. For localization, in addition to GPS, the inertial measuring unit is also used as a backup.

## Results

### Laboratory Testing

Ahead of a long-term deployment in a remote environment, we aim to run as many mission scenarios as possible in laboratory and outdoor environments in a controlled manner that will enable us to understand the performance capabilities and limitations of the SAM instrument. Since many parts of the instrument include elements such as metals, cables, foam, and electronic boards, it becomes critical to understand and have an idea of what to anticipate with the prolonged exposure to water, ions, and corrosive sulfur and nitrogen gases. The current prototype of the SAM instrument was only fully tested in the laboratory environment at the time of submitting this paper, although parts of this concept have already been tested in field campaigns in Boulby Mine and Movila Cave. Future long-term deployments in corrosive environments, namely, brine in Boulby mine, UK, and sulfidic water in Movile cave, Romania, will assess the long-term exposure’s detrimental effects. We anticipate some wearing in the steel parts, particularly with rust formation, which will provide feedback on the necessary design modifications required for long-term deployments in such extreme environments.

**Figure 3:**
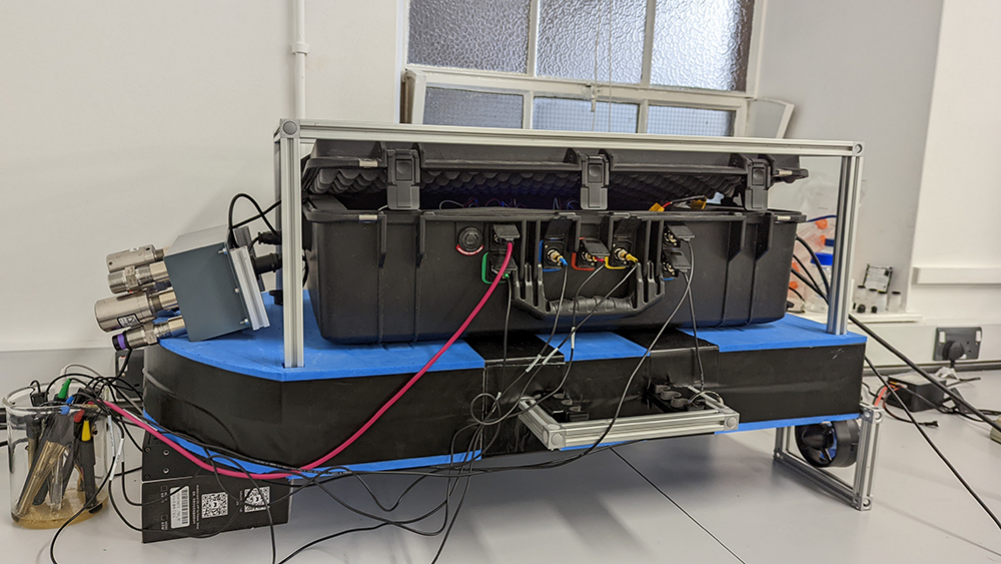
SAM instrument during a laboratory test under controlled conditions using brines from Boulby Mine. This test was conducted at the University of Aberdeen, UK, in July 2022.

**Figure 4:**
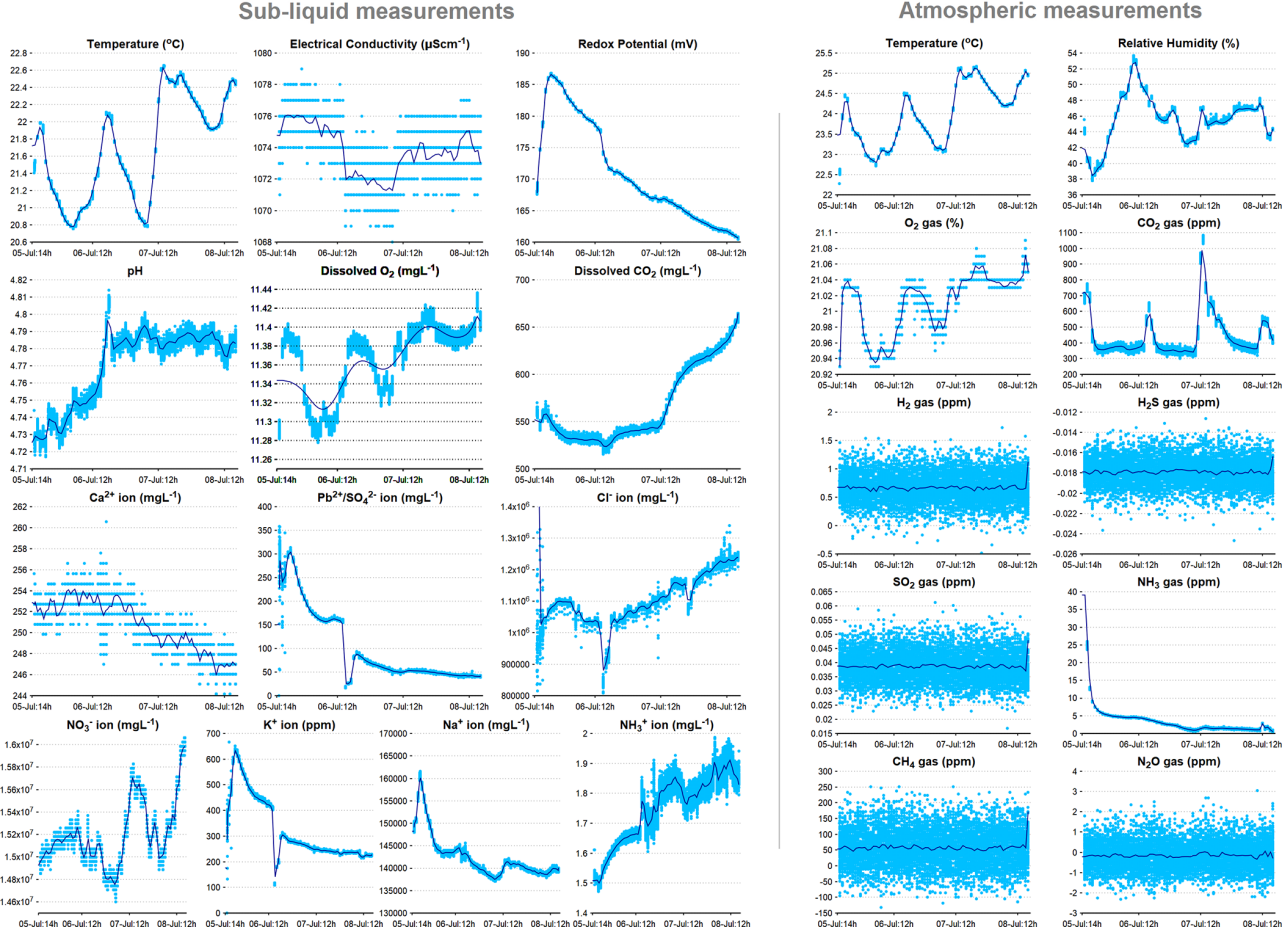
Sample data collected for about 3 days during the laboratory test conducted at the University of Aberdeen, UK, in July 2022, showing the liquid and gas measurements between July 5, 14:51:00 and July 8, 16:04:00. The liquid that is used for this laboratory test is from the brine pool in the Boulby mine, UK.

Figure 3 shows one of the testing configurations of the SAM instrument in the laboratory setting. The sub-liquid sensors are submerged in brine from the Boulby mine, UK, inside the glass beaker, while the system measures its properties for about 3 days. The sub-liquid sensors were removed from their standard configuration (on either side of the floating platform) and put in the glass beaker. The length of the cables of the sub-liquid sensors is nominal (about 1 m), and the gas sensors were installed at their nominal position. The data were collected for the first 5 min of every hour at 1 Hz, which provided 300 data points for an hour. The system is set up to wake up 2 min before the nominal measurements to warm up (the carbon dioxide sensor poses requirements of a warming up time of 10 s to reach a stable reading) or to stabilize the reading (the dissolved oxygen sensor requires 5–30 s to reach a stable reading) of some of the sensors. The system is set to sleep mode for the remaining 53 min of the hour to save battery power. Figure 4 shows the data of the liquid and gas measurements collected during this laboratory test.

Since the gas sensors were measuring the ambient laboratory atmosphere during this test, a nominal derivative performance of the SAM instrument can be concluded by comparing the measurements with the standard laboratory gas concentrations. With this approach, the oxygen (∼21%) and carbon dioxide (∼300–400 ppm nominal) gas concentrations were well in accordance with the standards within a closed indoor environment with sufficient ventilation. Since there is no major alteration in the test conditions with the exception of the natural diurnal cycle, some of the measurements, such as temperature and relative humidity, have varied naturally throughout the test. Some other measurements have shown gradual temperature-induced changes (mainly the liquid measurements, as they are prone to adjust to the air temperature because of the open system, with a clear indication in dissolved oxygen, lead/sulfate, nitrate, and sodium ion concentrations). Some other measurements (redox potential, carbon dioxide, and ammonia) have shown a gradual transition to nominal values after the operator exits the laboratory and the measurements stabilizing after a specific time (in the order of 2–3 h) to achieve equilibrium. Hence, it may be wise to neglect the first 2–3 h of initial data from the final analysis to draw better scientific conclusions. The general trend in Figure 4 shows a constant exchange of dissolved gases (diurnal gain and loss of oxygen from the liquid and carbon dioxide transfer from the atmosphere to the liquid). Most of the ion concentration measurements showed either an increasing or a decreasing trend. Of particular interest, the cations (calcium, lead, potassium, and sodium) showed a decreasing trend, whereas the anions (chloride and nitrate) showed an increasing trend. Other important measurements, such as electrical conductivity and pH, remained stable for the most part of the test. The sudden dip in the lead/sulfate, chloride, and potassium ion concentrations around 12:00 local time on July 6, 2022 is believed to be a temporary artifact due to moving the glass beaker containing the liquid. Since the trace gases such as hydrogen, hydrogen sulfide, sulfur dioxide, ammonia, methane, and nitrous oxide are not expected in good concentrations in the laboratory environment, the measurements returned were ideally in the range of zero offset values with large fluctuations.

### Floatability and Maneuverability Testing

The two independent operations of floatability and the maneuverability of the platform on which the SAM instrument is housed were tested in two different field campaigns: (i) natural brine pool at the Boulby mine, UK, during December 2022 and (ii) sulfidic lake in Mangalia, Romania during October 2022.

#### Site Description

Boulby mine (54.5561°N, 0.8234°W) is a deep underground salt mine located on the northeastern coast of England in the North Yorkshire region. Boulby mine is formed from a ∼250 million years old Permian Sea evaporite that hosts the Boulby Underground Laboratory at a depth of 1.1 km.^32^ The sulfidic lake (43.8287°N, 28.5695°E) is in the district of Mangalia, located in the eastern part of Romania along the Black Sea coast. The water in the lake is sourced from the backwaters of the Black Sea, whose excess sulfur content gives its black color and pungent odor.

**Figure 5:**
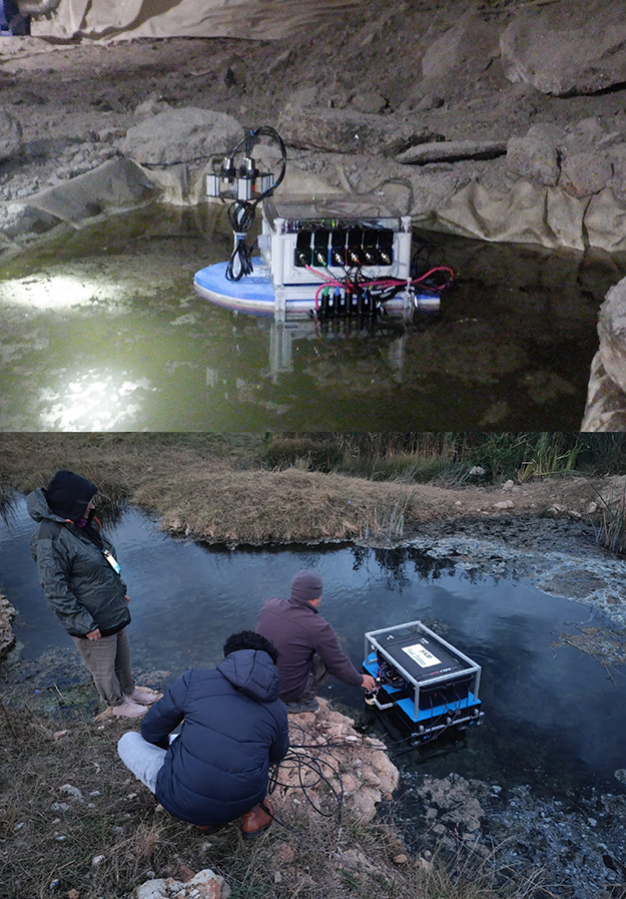
(top) Testing the pilot prototype of the SAM instrument during a field campaign in a natural brine pool at Boulby mine, UK, in December 2021. The campaign involved basic floatability testing of the platform material with some initial measurements of the brine for a week. (bottom) Testing the current prototype of the SAM instrument during a field campaign in a sulfidic lake at Mangalia, Romania, in October 2022. The campaign involved full floatability and maneuverability testing of the platform for an hour.

Figure 5 describes about the field campaigns for testing the pilot and current prototypes of the SAM instrument. While in the brine pool at the Boulby mine, the high-density polyethylene (HDPE) foam used as the floating material was tested as a part of the pilot prototype of the SAM instrument with some initial measurements (shown at the top of Figure 5), the field operation of the thrusters and the overall performance of the floating platform (floatability and the maneuverability) with the current prototype of the SAM instrument (shown in the bottom of Figure 5) was tested in the sulfidic lake in Mangalia, Romania.

The results for both test campaigns were successful. The floatability performance of the pilot prototype of the SAM instrument during the first campaign in the brine pool at Boulby mine, UK, was instrumental in designing the current prototype of the SAM instrument that was tested in the sulfidic lake in Mangalia, Romania. The performance of the HDPE foam chosen as the platform’s floating material was satisfactory, with the capability to carry the entire payload of the SAM instrument, including batteries (weighing around 10 kg), with little to no adverse chemical reaction from the brine or sulfur-rich water. This was indicative of the water line observed (not shown in the figure) during the campaign in the sulfidic lake in Mangalia, Romania. The water line was well below the halfway mark in the height axis of the floating platform (perpendicular to the surface of the liquid body), suggesting room for more payload in the future (at least additional 5 kg).

### Field Data Collection

The natural brine pool at the Boulby mine, UK, represents the only field campaign conducted so far to test the performance of the payload (sub-liquid and gas sensors) and evaluate the validity of the measurements obtained with the SAM instrument.

**Figure 6:**
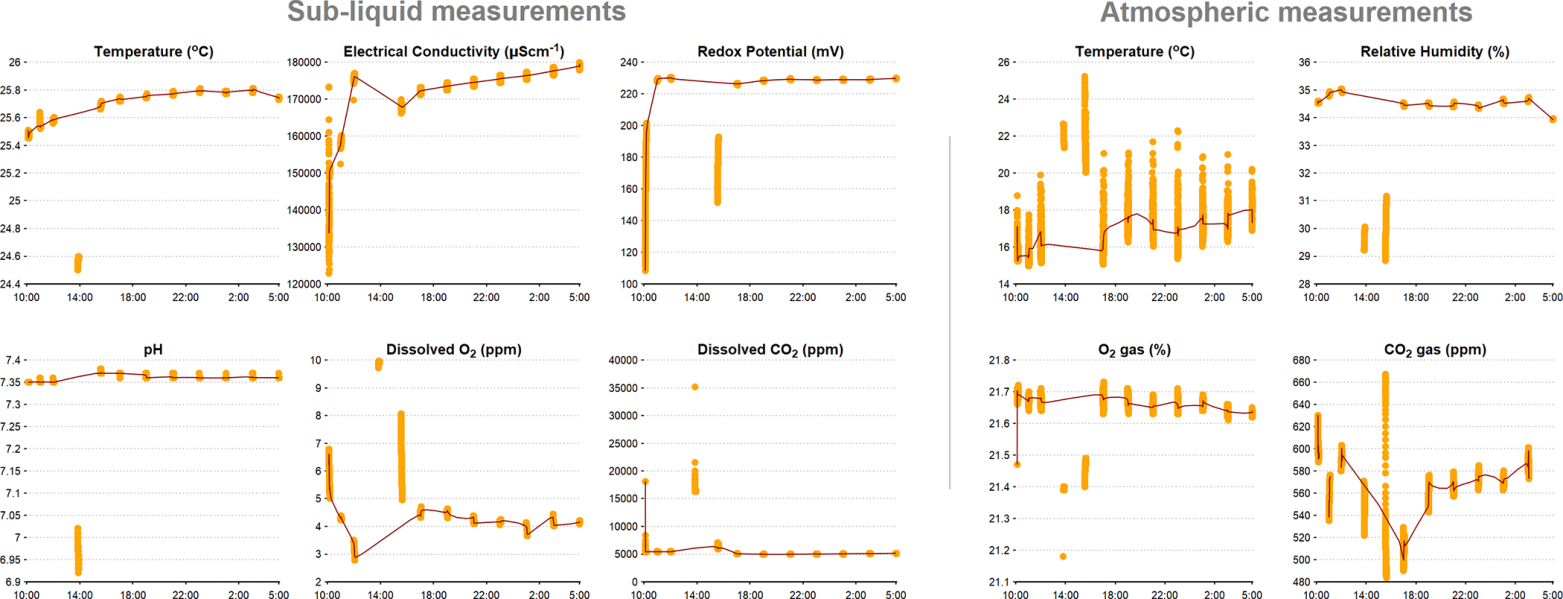
Sample data collected using the pilot prototype of the SAM instrument for 20 h during the field campaign in the natural brine pool at the Boulby mine, UK, in December 2021 between December 3, 10:00:00 and December 4, 05:05:00. The large variation between 14:00 and 16:00 is because of the sudden change in temperature of the air being pumped into the Mine from the surface. All other liquid and atmospheric properties show some temperature-induced effect.

For this campaign, which was conducted in December 2021, a pilot prototype of the SAM instrument (shown in Figure 5, top) was used. It comprises some of the measurements in the current prototype, including sub-liquid: temperature, electrical conductivity, oxidation–reduction potential, pH, dissolved oxygen, and dissolved carbon dioxide concentrations; atmosphere: temperature, relative humidity, oxygen, and carbon dioxide gas concentrations. Figure 6 shows the data of the liquid and gas measurements collected during the field campaign in the Boulby mine, UK.

The measurement shows the data collected over 20 h between December 3, 2021, 10:00:00 and December 4, 2021, 05:05:00. Since the environment in the mine is artificial, meaning the air is pumped from the surface through a tunnel and circulated throughout the mine, the measured parameters show sudden, unpredictable variations at 14:00 and 15:00, which are produced by the air circulation system. Figure 6 shows that the sub-liquid measurements of the brine were also affected by the changes in the atmosphere, which remained consistent throughout otherwise. A few distinguishable measurements of the natural brine are the electrical conductivity at ∼175,000 μS/cm or ∼17.5 S/m which is equivalent to ∼3 times the seawater conductivity (∼6 S/m) at the surface level,^33^ and dissolved oxygen at ∼4 mg/L or ppm, which is typically low at higher salt concentration.

## Discussion

The results presented here show the utility of the SAM instrument for monitoring various physicochemical parameters in the liquid and various gas concentrations in the aquatic ecosystems’ atmosphere to help determine the biogeochemical cycles between liquid and gas phases induced by microbes and industrial processes using commercially available sensors. The electronics and embedded microcontroller enabling the LoRa WAN network could read all the sensors and transmit data wirelessly between the float and surface stations, while leaving a local copy of the data in MicroSD card on both stations as a backup. The dimensions of the SAM instrument, including the floating platform, are 1045 mm length × 795 mm width × 525 mm height; it has a mass of 14.9 kg and a maximum power consumption in laboratory test configuration of ∼2.5 and ∼209.5 W with thrusters. A fully charged (not charged during the operation) primary (lithium-ion) battery in the float station and the backup (lead acid AGM) battery were sufficient for a nominal test operation of 15 days with continuous hourly measurements. This fully integrated system should be valuable for others to conduct continuous water quality and atmospheric monitoring campaigns.

This is an open-source system, and we have provided schematics for others to replicate the data logger with the embedded MicroSD data storage and a preferred remote data transmission to laboratory servers. The various operational concepts will help decide the additional components needed to be installed depending on the deployment environment. For example, a solar charge controller and GSM or iridium network module will form the main electronics of the system for an open surface liquid body deployment. The circuit boards can be purchased, as they are factory-tested for water ingress and can be mounted directly on the circuit board with other components. No soldering is required. All other aspects of the construction involve mounting circuit boards in IP67-rated enclosures and wiring. In order to make the wiring flexible, all sensor connections are made with removable screw terminals and jumper terminals. We have also provided a computer-aided design file for the entire assembly with accurate dimensions, weight, and power consumption.

Due to its versatility and independent nature, the SAM instrument can be used to study natural aquatic systems with climate-impacting applications in remote locations with restricted access to power, data communication, and maintenance. Nitrogen deposition, one of the climate-changing phenomena, is associated with atmospheric methane and nitrous oxide emissions from wetlands^34^ and high-latitude warming, which have stimulated carbon dioxide and methane emissions from permafrost peatlands.^35^ Sulfur and ammonia gas cycling in niche ecosystems have been discovered to be associated with microbial diversity.^36^ Similarly, microbes can also be responsible for sulfur, carbon, and nitrogen cycling.^37,38^ Understanding the exchange of these greenhouse gases is vital for predicting the rate of progress of various climate-impacting processes that follow. By monitoring these gases continuously, some of the puzzles surrounding the contribution of these gases toward climate change may be solved.

Monitoring the physicochemical parameters can be beneficial for industrial applications too, such as in fish farms. Temperature, pH, dissolved oxygen, total suspended solids, ammonia, nitrite, and nitrate concentrations have to be continuously measured to evaluate the permissible levels for aquaponic systems that provide information about the survival, growth, and food intake of fish.^39,40^ Also, pH is an excellent indicator to observe the nutritional profile in freshwater fishery sectors, including calcium, magnesium, iron, manganese, and zinc,^41^ and monitoring the electrical conductivity, pH, and oxidation–reduction potential becomes vital in brackish water fish cultures.^42^ The same parameters can also be used to assess the quality of groundwater that could be compromised by anthropogenic activities,^43^ industrial effluents,^44,45^ and algal bloom contamination.^46,47^

Although the SAM instrument offers several research and industrial applications, the deployment and operation of the SAM instrument do come with risks and challenges. Some of the field sites, including saline lakes, may host salt crusty deposits on lake surface, making the floating platform’s maneuvering tricky. Several works have investigated and proposed solutions with the use of simple camera^16,19,23^ or ultrasound sensors^13^ for detecting and navigating around such obstructions. Long-term deployments also impose restrictions on recalibration due to possible degradation of the sensors (higher in a corrosive saline environment). According to the datasheets of the various sensors used in the SAM instrument, the time before recalibration is determined by the types of probes and the level of chemical reaction occurring in the deployed field environment. Because every case is different, there is no predefined schedule for recalibration. In environments that are mild (not too oxidating or acidic or salty), the probes need to be recalibrated once per year for the first 2 years and, after that, every ∼6 months. If the probes are used in an environment known to have multiple cations and anions inducing chemical reactions (such as saline lakes), the recalibration should be done monthly and before every deployment. However, the working life of the probes depends on the probe type, spanning between 2 years for glass electrodes (ORP, pH, etc.) to 10 years for metal electrodes (EC). For field deployments that require several months to years in a harsh environment, the degradation of the sensors needs to be taken into account. Some probes may stop working after the depletion of its electrolyte solution which may occur in 2 years or earlier (DO), while others may degrade with corrosion on its electrode surface (EC). In cases like this, it is best to limit the maximum duration of the field deployment to a year in clean environments such as freshwater lakes and ponds and to 6 months for saline environments.

Another important consideration during deployment of the SAM instrument in environments of ecological importance and pristine nature is microbiological contamination control. During the Minimally Invasive Direct Glacial Access project (MIDGE), IceMole, a maneuverable thermoelectric melting probe, was used to collect the first englacial brine samples from Blood Falls, Antarctica. A specialized protocol was followed to reduce the exterior bio-load by an order of magnitude, to levels standard in most clean rooms, and 1–3 orders of magnitude below that of Taylor Glacier ice surrounding Blood Falls, in order to maintain the scientific integrity of samples collected and minimize the impact to this specially protected ecosystem. Prior to deployment, the exterior surfaces of the IceMole were cleaned using 3% H_2_O_2_ and rinsed with Nanopure deionized water.^48,49^ Since the probes and sensors of the SAM instrument are only compatible with chemical sterilization, the same cleaning process with 3% H_2_O_2_ and deionized water should be used prior to deployment.

## Conclusions

This paper describes an open-source, autonomous, and modular system, the SAM instrument, to monitor the biochemistry of natural aquatic ecosystems. Throughout the design and development phase of the instrument, it was intended for use in remote field environments where traditional biochemical monitoring mechanisms with sophisticated and large equipment cannot be implemented. However, since we are in the early phase of the development, the SAM instrument and its associated systems are still too large to transport easily and portable only with a standard-size pallet across various field sites of interest to deploy. This particularly complicates matters when transporting across country borders with standard freight carriers that cause unnecessary delays and customs charges. In order to counter this, the future design of the SAM instrument will be miniaturized to accommodate inside a flight case for ease of carrying manually across borders and deployment in any field environment around the world.

A few other planned future improvements to the system include accessing various depths of the liquid body to obtain a vertical profile of stratified measurements. This will be achieved with the help of an underwater mechanism, lowered from the floating platform with the sub-liquid sensors or a maneuverable underwater probe that will carry the sub-liquid sensors to various depths while measuring. Potential additional features of the underwater mechanism/ maneuverable probe will include an autonomous liquid sampler as a first step to sample and store fresh liquid samples for biological analysis with a method that is yet to be finalized. Upon further improvements, the SAM instrument is expected to be tested in the laboratory and deployed in Movile cave, Mangalia, Romania, with long-term deployment goals in Iceland, Lake Magic in Western Australia, and Tso Kar Lake in Ladakh, India.

Such autonomous and modular systems are in high demand and can be used for long-term monitoring of the climate-impacting processes on Earth with proper adaptations in accordance with a suitable operations concept required for the application. Upon completion of the necessary design changes and end-to-end development and testing phases, the applications could also go beyond Earth to monitor, sample, and analyze extraterrestrial aquatic systems in the coming years with upcoming ambitious space missions to other ocean worlds of our solar system.^50^

## Supporting Information Available

SAM instrument sensor specifications; photographs of the sub-liquid and gas sensor circuits; circuit diagram for power source and distribution and thruster control; electronics design of the sub-liquid and gas sensors; sensor calibration scheme and standards used; computer-aided design model of the SAM instrument; and physical parameters of the SAM instrument (PDF)
