# Supplementary material for: Sub-Liquid and Atmospheric Measurement Instrument To Autonomously Monitor the Biochemistry of Natural Aquatic Ecosystems
Source: ACS ES T Water. 2023 Jun 22;3(8):2338–54. doi: 10.1021/acsestwater.3c00082 (PMC10425959; doi:10.1021/acsestwater.3c00082)
Supplement: Supplementary file 1 — ew3c00082_si_001.pdf [file ew3c00082_si_001.pdf]

Sub-liquid and Atmospheric Measurement (SAM) instrument to autonomously monitor the biochemistry of natural aquatic ecosystems

Miracle Israel Nazarious[1, *], Maria-Paz Zorzano[2], Javier Martin-Torres[1, 3]

[1]School of Geosciences, University of Aberdeen, Meston Building, King's College, Aberdeen AB24 3UE, UK

[2]Centro de Astrobiología (CSIC-INTA), Torrejon de Ardoz, 28850 Madrid, Spain

[3]Instituto Andaluz de Ciencias de la Tierra (CSIC-UGR), 18100 Granada, Spain

Supporting Information

SAM Instrument Sensor Specifications

Table S1 elaborates on the selected sensors' specifications (measurement range, accuracy, resolution) and their nominal operating temperature and pH ranges wherever applicable.

Table S1. Summary of sensor parameters used in the SAM instrument

Measured parameter	Manufacturer	Sensor	Measurement range	Accuracy	Resolution	
**Sub-liquid Temperature (T_{liquid})**	Atlas Scientific	PT-1000 Temperature Probe in 100mm Temperature Thermowell with EZO™ RTD Circuit	-200 °C – 850 °C	± (0.15 + (0.002*t))	-	
**Pressure (P)**	Atlas Scientific	EZO-PRS™ Embedded Pressure Sensor	0 – 50 psi<br>or<br>0 – 344738 Pa	±2% (1 psi)	0.001	
**Air Temperature (T_{air})**	Atlas Scientific	EZO-HUM™ Embedded Humidity Probe			-	
**Relative Humidity (RH)**			0 – 100%	±2%	-	
**Electrical Conductivity (EC)**	Atlas Scientific	Conductivity Probe K 10 with EZO™ Conductivity Circuit	0.07 − 500,000+ µS/cm	±2%	0.07 – 99.99	0.01 µS/cm
			T = 1 − 110 °C			
			P_{max} = 200 psi		100.1 – 999.9	0.1µ S/cm
			d_{Max} = 141m (463 ft)			
					1,000 – 9,999	1.0µ S/cm

					10,000 – 99,990	10µS/cm
					100,000 – 999,900	100 µS/cm
**Oxidation-Reduction Potential (ORP)**	Atlas Scientific	Lab Grade ORP Probe with EZO™ ORP Circuit	-2000mV – 2000mV<br>T: 1 – 99 °C<br>P_{max}: 100 psi<br>d_{max}: 70m (230 ft)	±1mV	-	
**pH**	Atlas Scientific	Lab Grade pH Probe with EZO™ pH Circuit	0.001 – 14.000<br>T: -5 – 99 °C<br>P_{max}: 100 psi<br>d_{max}: 70m (230 ft)	±0.002	.001	
**Calcium (Ca^{2+})**	Hanna Instruments	HI-4104 Calcium combination ISE electrode with Atlas Scientific EZO™ ORP Circuit	0.12 – 40080 ppm<br>pH: 4 – 10<br>T: 0 – 40°C	±1mV	-	
**Lead/Sulfate (Pb^{2+}/SO_4^{2-})**	Hanna Instruments	HI-4112 Lead/Sulfate combination ISE electrode with Atlas Scientific EZO™	0.21 – 20700 ppm<br>pH: 4 – 7<br>T: 0 – 80°C	±1mV	-	

		ORP Circuit			
**Chloride (Cl^-)**	Hanna Instruments	HI-4107 Chloride combination ISE electrode with Atlas Scientific EZO™ ORP Circuit	1.8 – 35000 ppm<br>pH: 2 – 11<br>T: 0 – 80°C	±1mV	-
**Nitrate (NO_3^-)**	Hanna Instruments	HI-4113 Nitrate combination ISE electrode with Atlas Scientific EZO™ ORP Circuit	0.62 – 6200 ppm<br>pH: 3 – 8<br>T: 0 – 40°C	±1mV	-
**Potassium (K^+)**	Hanna Instruments	HI-4114 Potassium combination ISE electrode with Atlas Scientific EZO™ ORP Circuit	0.039 – 39100 ppm<br>pH: 1.5 – 12<br>T: 0 to 40°C	±1mV	-
**Sodium (Na^+)**	Hanna Instruments	FC300B Sodium ion electrode with Atlas Scientific EZO™ ORP Circuit	0.039 – 39100 ppm<br>pH: 9.75 – 14<br>T: 0 to 80°C	±1mV	-
**Dissolved Oxygen (DO)**	Atlas Scientific	Lab Grade Dissolved Oxygen Probe with	0.01 − 100+ mg/L<br>or	±0.05 mg/L	-

		EZO™ Dissolved Oxygen Circuit	0.1 − 400+ %<br>T: 1 − 60 °C<br>P_{max}: 500 psi<br>d_{max}: 352m (1,157 ft)		
**Dissolved Carbon dioxide (DCO_2)**	Hanna Instruments	HI-4105 Carbon dioxide combination ISE electrode with Atlas Scientific EZO™ ORP Circuit	4.4 – 440 ppm<br>pH: 4.2 – 5.2<br>T: 0 to 40°C	±1mV	-
**Dissolved Ammonia (NH_3^+)**	Hanna Instruments	HI-4101 Ammonia combination ISE electrode with Atlas Scientific EZO™ ORP Circuit	0.02 – 17000 ppm<br>pH: >11<br>T: 0 to 40°C	±1mV	-
**Oxygen (O_2)**	Atlas Scientific	EZO-O2™ Embedded Oxygen Sensor	0 – 42%	±0.01%	0.01
**Carbon dioxide (CO_2)**	Atlas Scientific	EZO-CO2™ Embedded Carbon Dioxide Sensor	0 − 10000 ppm	(±5%) + (±50 ppm)	1 ppm
	Dynament	Platinum Series Carbon Dioxide Sensor	0 – 10000ppm – 5%	±2%	0.01%

**Methane (CH_4)**	Dynament	Platinum Series Hydrocarbon Sensor	0 – 5 – 100%	±2%	0- 5%	0.01 %
					5-100%	0.1%
**Nitrous Oxide (N_2O)**	Dynament	Platinum Series Nitrous Oxide Sensor	0 – 1000 ppm – 1%	±5%	0-1000 ppm	20 ppm
					1000 ppm – 1%	0.01 %
**Hydrogen (H_2)**	Alphasense UK	H2-BF Hydrogen Sensor with Individual Sensor Board (ISB)	0 – 5000 ppm	±15 ppm	< 0.8 ppm	
**Hydrogen Sulfide (H_2S)**	Alphasense UK	H2S-BE Hydrogen Sulfide Sensor – High Concentration with Individual Sensor Board (ISB)	0 – 2000 ppm	+/±ppm	< 0.5 ppm	
**Sulfur dioxide (SO_2)**	Alphasense UK	SO2-BE Sulfur Dioxide Sensor - High Concentration with Individual Sensor Board (ISB)	0 – 2000 ppm	±2 ppm	< 2 ppm	

**Ammonia (NH_3)**	Alphasense UK	NH3-B1 Ammonia Sensor with Individual Sensor Board (ISB)	0 – 100 ppm	±10 ppm	-

Electronics

The primary electronics layout of the SAM instrument is shown in Figure S1. The main components of the platform electronics include the signal processing circuits for the sub-liquid (blue) and gas sensors (grey), power source and distribution (red), and circuits for operating and controlling the thrusters (yellow). The electronics for the sub-liquid sensors are mounted on a laser-cut black Polyethylene (PE) sheet and housed in a Peli 1605 Air Case (Peli Products UK), made of HPX^2-super-lightweight resin offering IP67-rated protection and padded with foam. Similarly, the electronic components for the power source, distribution, and thruster control are also housed in the same case. However, the electronics for gas sensors are housed in an individual stainless-steel casing (RS PRO) with holes drilled on its surface according to the dimensions of the sensing area of the gas sensors.

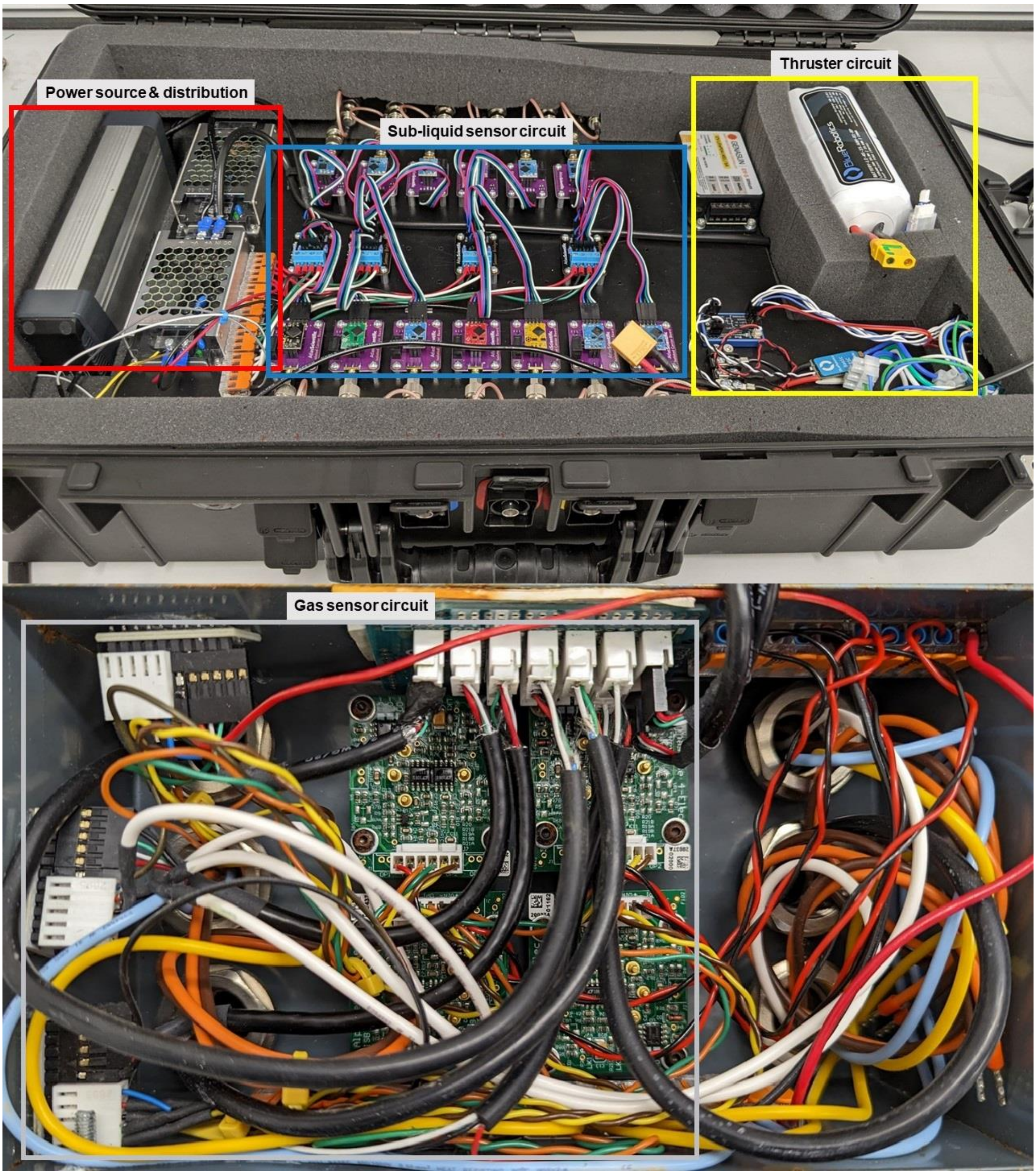

Figure S1. SAM instrument electronics (clockwise): (a) Power source and distribution circuit (red), (b) Circuit board and connections for the sub-liquid sensors (blue), (c) Thruster circuit (yellow), (d) Circuit board and connections for the gas sensors (grey).

Power source and distribution: A 200W (52Ah) Lithium-ion Powerbank (Mobile Solar Chargers, UK) is used as an internal power source with an option to route the 12V/5A output from the power bank to either a 12→5 V DC converter (Mean Well) or a 12→3.3 V DC converter (Mean Well) depending on the power rating of the sensor circuits being used. The 5/3.3 V DC that is supplied and distributed powers the main computer of the float station and the electronic circuits (Atlas Scientific, US) responsible for the functioning of the sub-liquid and gas sensors. In the open surface environment operational configuration (more information

in "Operational concept" section) of the SAM instrument, the power bank will be recharged with a 60W Monocrystalline ETFE folding solar panel with charger (Mobile Solar Chargers, UK) that is installed flat over the floating platform. Figure S2 (left) shows the power source and distribution circuit connection. The circuit's primary connection comprises the power supply's 12 VDC and ground signals, colour-coded as red and black, respectively.

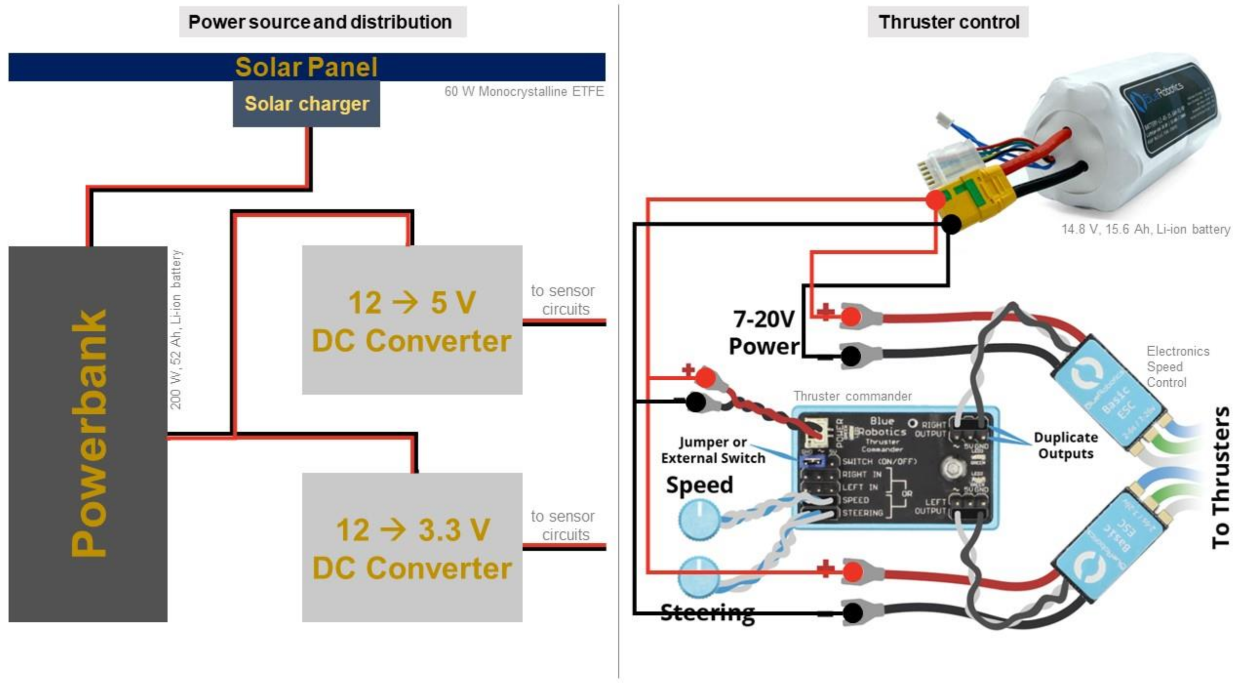

Figure S2. (left) Power source and distribution circuit, (right) thruster control circuit.

Thruster control: A separate 4S Lithium-ion battery (14.8V, 15.6 Ah, Blue Robotics) is dedicated to power the thruster control circuit. An external switch is connected to activate the circuit before deployment manually. The thruster commander supports the electronic speed control (one for each thruster) that controls the voltage level and, thereby, the speed of the motors, using individual knobs for speed and steering. The cable length from the thruster commander to the knobs can be extended to house the control board with the surface station. Figure S2 (right) shows the circuit connection for thruster control. The circuit's primary connection comprises the power supply's 12 VDC and ground signals, colour-coded as red and black, respectively. In place of manual knob controls, a remote transceiver can also be used to control the speed and steering of the thrusters.

Signal processing

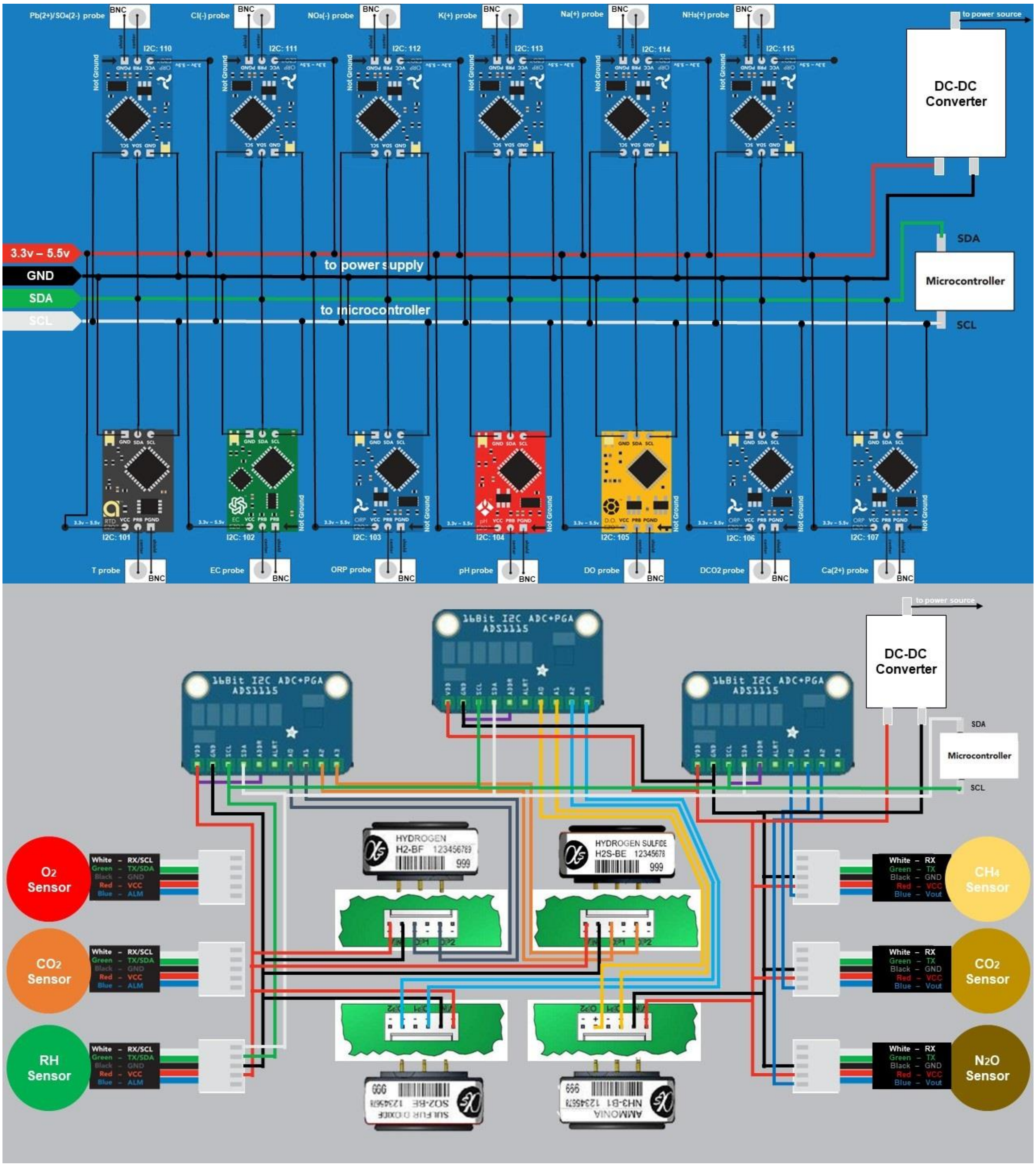

Figure S3. (top) Schematic of the circuit and signal connections of the sub-liquid sensors, (bottom) Schematic of the circuit and signal connections of the gas sensors.

The power supply and data communication signals from various sub-liquid and gas sensors to the main computer are shown. Figure S3 (top) illustrates the signal processing connections for the sub-liquid sensors.

The primary connections in both the circuits comprise the 3.3 – 5 VDC and ground signals of the power supply, colour-coded as red and black respectively, and the data (SDA) and clock (SCL) signals of the I^2C data communication protocol, colour-coded as green and grey

respectively. Each of the 13 EZO™ circuits (Atlas Scientific) used for the sub-liquid sensors has these common connections which are mapped using a series of sensor bridges (not shown in the figure) for ease of tidying the connecting wires. The power supply lines are sourced to either the 12→5 V DC or the 12→3.3 V DC converter, depending on the power rating of the sensor circuits. The EZO™ circuits can operate with both the 3.3 V and 5 V DC voltage ranges, but since some of the gas sensor boards can only operate at a 5 V DC voltage level, the entire signal processing circuit for sub-liquid and gas sensors used a 5 V DC power supply provided by the 12→5 V DC converter. The data and clock signals of the I^2C data communication protocol are directly connected to the microcontroller (Arduino MKR family), which runs at 3.3 V DC by feeding the power from the same 5 V DC from the 12→5 V DC converter into the V_{in} pin of the microcontroller board that regulates the voltage to 3.3 V DC level. The 13 EZO™ circuits also provide a 2-pin connection through a BNC for signal inputs from the sub-liquid sensors that measure various physicochemical parameters in the liquid. The circuit uses dedicated EZO™ circuits for connecting to temperature (T), electrical conductivity (EC), oxidation-reduction potential (ORP), pH and dissolved oxygen (DO) probes and calibrated to the absolute values of their corresponding measurements while for measuring dissolved carbon dioxide, and ions such as calcium, lead/sulphate, chloride, nitrate, potassium, sodium and ammonia, their respective ion selective electrodes (ISEs) are calibrated with the EZO™ ORP circuit.

Figure S3 (bottom) illustrates the connections for the gas sensors. The power supply and data communication signals are tapped into the sub-liquid sensors' signal processing circuit through one of the ports in the sensor bridge. Some gas sensors (Alphasense UK and Dynament), unlike the EZO™ circuits, produce an analog voltage output signals (through Individual Sensor Board, ISB for Alphasense UK sensors and inbuilt circuit for Dynament sensors) that are equivalent to the gas concentration. These analog voltage output signals from the gas sensors are read using the 4-channel 16-bit Analog to Digital Converter (ADS1115, Texas Instruments) that can read up to four analog voltage outputs from the gas sensors (each Alphasense UK gas sensors have a working electrode and an auxiliary electrode whose analog voltage output has to be read) and communicate with the microcontroller using the I^2C data communication protocol. Only the Atlas Scientific gas and relative humidity sensors communicate directly with the microcontroller using the I^2C protocol.

Sensor Calibration

Table S2 provides the calibration scheme for the sub-liquid sensors and their circuits.

Table S2. Calibration scheme of the sub-liquid sensors

Measurement	Circuit to be calibrated	1/2/3- point calibration	Calibration standards
**Temperature (T)**	EZO™ RTD Circuit	1	RS PRO RS-91 Mini Temperature and Relative Humidity Datalogger (20 – 60 °C, Accuracy: ±0.5 °C for 0 – 40 °C, ±1 °C for -20 – 0 & 40 – 60 °C, Resolution: 0.1 °C)

**Electrical Conductivity (EC)**	EZO™ Conductivity Circuit	2	Low: 12,880 µS/cm, High: 150,000 µS/cm
**Oxidation Reduction Potential (ORP)**	EZO™ ORP Circuit	1	225 mV
**pH**	EZO™ pH Circuit	3	Low: 4.00, Mid: 7.00, High: 14.00
**Dissolved Oxygen (DO)**	EZO™ Dissolved Oxygen Circuit	1	0 mg/L
**Dissolved Carbon dioxide**	EZO™ ORP Circuit	1	225 mV
**Calcium (Ca^{2+})**	EZO™ ORP Circuit	1	225 mV
**Lead/Sulfate (Pb^{2+}/SO_4^{2-})**	EZO™ ORP Circuit	1	225 mV
**Chloride (Cl^-)**	EZO™ ORP Circuit	1	225 mV
**Nitrate (NO_3^-)**	EZO™ ORP Circuit	1	225 mV
**Potassium (K^+)**	EZO™ ORP Circuit	1	225 mV
**Sodium (Na^+)**	EZO™ ORP Circuit	1	225 mV
**Ammonia (NH_3^+)**	EZO™ ORP Circuit	1	225 mV

CAD Model of SAM Instrument

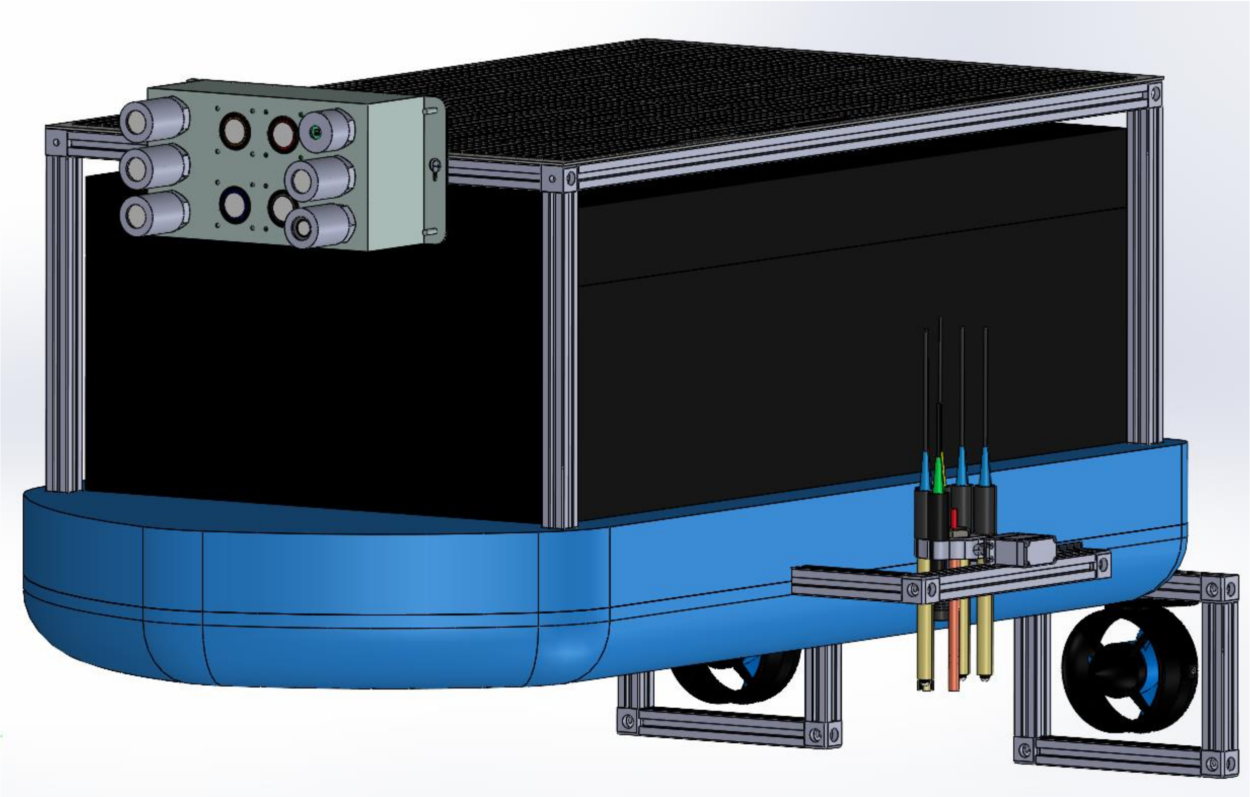

Figure S4. Computer-aided design model of the SAM instrument.

Physical Parameters of SAM Instrument

Table S3. Parameters of the SAM instrument including the physical dimensions, weight and power consumption.

Parameter	Payload	Platform	Overall
**Physical Dimensions (L × W × H in mm)**	NA	1045 × 795 × 525	1045 × 795 × 525
**Weight (kg)**	7.5	7.4	14.9
**Maximum Power Consumption (W)**	~2.5 (primary + backup battery)	207 (thruster battery)	~209.5
